# Effect of complementary foods fortified with *Moringa oleifera* leaf powder on hemoglobin concentration and growth of infants in the Eastern Region of Ghana

**DOI:** 10.1002/fsn3.890

**Published:** 2018-11-20

**Authors:** Laurene Boateng, Wilhemina Quarpong, Agartha Ohemeng, Matilda Asante, Matilda Steiner‐Asiedu

**Affiliations:** ^1^ Department of Nutrition and Food Science University of Ghana Legon Ghana; ^2^ Department of Dietetics University of Ghana Accra Ghana

**Keywords:** complementary foods, low resource settings, malnutrition, *Moringa oleifera* leaf powder

## Abstract

Complementary foods that are deficient in both macronutrients and micronutrients coupled with a high burden of infections during the complementary feeding period are major underlying causes of child malnutrition in developing countries. Among the recent efforts to combat malnutrition by improving the quality of complementary foods in the developing world is the use of *Moringa oleifera* leaf powder (MLP) as a food fortificant. We conducted a randomized controlled trial to test the effect of feeding MLP‐fortified complementary food on hemoglobin concentration and growth of infants and young children after 4 months of feeding. Infants aged 8–12 months were randomly assigned to receive one of three study foods; Weanimix a cereal‐legume blend formulated with *Moringa* (*MCL‐35g*), MLP sprinkled on infants’ usual complementary foods (*MS‐5g*) and the control food Weanimix without *Moringa* (*CF‐35g*). Blood samples for hemoglobin determination as well as dietary intake and anthropometric data were collected at baseline and endline for 237 infants who completed the study. Data analysis was performed with SPSS (version 20) and comparisons were done by analysis of covariance (ANCOVA). There were no significant differences in hemoglobin concentration or growth indicators at endline between the three study groups. Findings of this study indicated that feeding infants a 5 g daily dose of MLP, either as part of a cereal‐legume blend or as a supplement which was sprinkled on infants’ usual complementary foods for 4 months, did not significantly improve infants’ hemoglobin concentration or growth indicators.

## 1 INTRODUCTION

The complementary feeding period is generally associated with considerable growth faltering in low‐income populations (Victora, de Onis, Hallal, Blossner, & Shrimpton, 2010). In developing countries, complementary foods that are deficient in both macronutrients and micronutrients coupled with a high burden of infections during the complementary feeding period are major underlying causes of child malnutrition. In Ghana, the recent demographic and health survey (DHS) data showed that 19% and 5% of children under 5 years old are stunted and wasted, respectively (Ghana Statistical Service (GSS), Ghana Health Service (GHS), and ICF International, 2015). The survey results further revealed that micronutrient malnutrition is still highly prevalent and persistent with 66% of children under 5 years suffering from varying degrees of anemia.

One major cause of micronutrient malnutrition in low‐income populations is the lack of access to a variety of foods (Miller & Welch, 2013). Improving the quality of complementary foods is reported to be one of the most cost‐effective approaches for reducing morbidity and mortality in young children and improving their health (Krebs & Hambidge, 2007) and there is a renewed emphasis on the use of locally available food ingredients for improving complementary foods (Babu, 2000).

*Moringa oleifera*, a tree found in abundance in the dry tropics, is reported to be a rich source of proteins and micronutrients. The leaves of *Moringa oleifera* could be harvested and cheaply dried with solar dryers and milled to form a powder that could be stored for use in rural households (Glover‐Amengor, Aryeetey, Afari, & Nyarko, 2017). Among the recent efforts to combat malnutrition by improving the quality of complementary foods in the developing world is the use of *Moringa oleifera* leaf powder (MLP) as a food fortificant (Oyeyinka & Oyeyinka, 2018). To date, however, studies on the suitability of MLP as an ingredient in complementary foods have focused on the proximate composition, sensory analysis, microbiological safety of the foods formulated with MLP and a few observational studies on its potential to improve nutritional status of infants in the first 1000 days of life. Trials demonstrating its long‐term acceptability and efficacy when incorporated in complementary foods lacking (Glover‐Amengor et al., 2017), although its high protein and micronutrient composition suggest that it may be an effective treatment against malnutrition. The aim of this study was to test the effect of feeding complementary food to which MLP has been added on hemoglobin concentration and growth of infants and young children in Ghana, a developing country. We formulated two hypotheses. Firstly, that infants fed MLP leaf powder as part of a cereal‐legume blended flour for 4 months would have significantly higher hemoglobin levels and weight and length gain than infants fed the cereal‐legume blended flour without MLP after 4 months of feeding. We further hypothesized that infants fed MLP as a supplement to be sprinkled on their usual diets would have significantly higher hemoglobin concentrations than infants fed the cereal‐legume blended flour without MLP, after 4 months of feeding.

## 2 MATERIALS AND METHODS

### 2.1 Study site and population

The study was carried out in the Upper Manya Krobo district of the Eastern Region of Ghana. The Eastern region registered the highest rates of stunting and anemia among infants and young children in the demographic and health survey (Ghana Statistical Service (GSS), Ghana Health Service (GHS), and ICF International, 2009). Agriculture is the main economic activity of the people of the district, employing about 80% of the population, most of whom are subsistence farmers with very few commercial ones. The study population included infants under two years of age attending weight monitoring sessions at Maternal and Child Health facilities in the Upper Manya Krobo District. Inclusion criteria for infants were as follows: a child must be aged between 8 and 12 months at the time of recruiting; must be breastfeeding; have no congenital abnormalities; have been assigned maternal and child health cards; and whose mothers or caregivers planned to stay at the study site for the duration of the study were eligible for enrollment in the study. Infants who did not meet these criteria were excluded from the study. Additionally, infants who had known intolerances to any of the ingredients of the study foods were excluded from the study. The trial was registered retrospectively with the ISRCTN registry with reference number—ISRCTN14377902.

### 2.2 Study design

The study was a cluster randomized controlled trial with 3 arms. The first arm received MLP as part of a cereal‐legume blended flour (*MCL‐35g*). The second arm (*MS‐5g*) received *Moringa* as “sprinkles” (a food supplement) to be added to the usual diets and the third arm (*CF‐35g*) which was used as the control arm received the cereal‐legume blend complementary food Weanimix without MLP. The ingredient composition of the foods ate is described in Table 1. The study foods were hygienically produced and packaged at the Food Research Institute in Accra. Nutritional composition of study foods is reported in Table 2. Acceptability of the study foods was tested and found to be acceptable ahead of the study (Boateng, Nyarko, Asante, & Steiner‐Asiedu, 2018).

**Table 1:** Ingredient composition of study foods

Ingredients	Composition of study foods		
	*CF‐35g* (control) daily ration size—35 g	*MS‐5g* daily ration size—5 g	*MCL‐35g* daily ration size—35 g
Maize (g)	26	‐	21
Soybean (g)	9	‐	9
*Moringa* leaf powder—MLP (g)	‐	5	5
	35 g	5 g	35 g

*Note*

*CF‐35g—*control; *MCL‐35g—Moringa* with *Weanimix; MS‐5g—Moringa* as Sprinkles.

**Table 2:** Energy and nutrient composition per 100 g [per daily ration size] of study foods

Parameter	*CF‐35g*	*MCL‐35g*	*MS‐5g*
Crude protein (%)	16.80 [5.88]	18.29 [6.40]	27.8 [1.39]
Crude fat (%)	8.17 [2.86]	8.94 [3.13]	5.75 [0.29]
Ash (%)	2.12 [0.72]	2.78 [0.97]	8.31 [0.42]
Crude fiber (%)	1.84 [0.64]	1.67 [0.59]	5.39 [0.27]
Carbohydrates (%)	66.35 [23.22]	64.91 [22.72]	10.51 [0.53]
Energy (kcal)	406.13 [142.15]	413.26 [144.64]	205.00 [10.25]
Vitamin A (μgRE/100 g)	0.78 [0.27]	148.43 [51.95]	496.4 [24.82]
Iron (mg/100 g)	7.25 [2.54]	10.81 [3.78]	22.6 [1.13]

*Note*

*CF‐35g—*control; *MCL‐35g—Moringa* with *Weanimix; MS‐5g—Moringa* as Sprinkles.

### 2.3 Sample size

The primary outcome was hemoglobin concentration after 4 months of feeding. Growth (weight and length gain) was the secondary outcome. Sample size calculation was based on the detection of differences among the three study groups equivalent to a “medium” effect size [Cohen's *d =* (difference/pooled *SD*) = 0.5] (Cohen, 1988). With a type I error of 0.05 and a 0.8 probability of detecting a true difference (1−*β*), the required sample size per group was 77. Allowing for 15% attrition in the three groups, the target sample size for each group was 91 giving a total of 273 study participants. Multiplying our sample size by a factor of 1.5 to account for the cluster effect resulted in a total of 411 infants, 137 per each study group.

### 2.4 Procedures

Study infants were recruited from the seven out of the eight primary health centers (PHCs) in the Upper Manya Krobo (UMKD) district and their catchment communities. The PHC was the unit of randomization, and each PHC and its catchment communities were allocated to one study food by simple randomization. Infants were recruited after consent had been obtained from their mothers or caregivers and assigned to receive one of the three study diets based on the PHC from where the infant was recruited. There was no placebo group because of ethical concerns about providing no treatment to infants who may have tested low on measures (e.g., hemoglobin) at baseline. The feeding period lasted for 16 weeks for each infant. The trial could not be blinded because the study foods were very dissimilar in appearance.

### 2.5 Pre‐intervention phase

Mothers and caregivers of recruited infants were visited at home to verify eligibility, explain the study protocol in detail, and obtain written informed consent. Background information, anthropometry, 24‐hr recalls and hemoglobin levels were collected at baseline.

### 2.6 Intervention phase

Study foods were delivered to the infants on a bi‐weekly basis. Cooking demonstrations were carried out at the PHCs and in the catchment communities to teach mothers how to prepare study foods. Each child in study group 1 (*MCL‐35g*) and study group 3 (*CF‐35g*) received 35 g of study food per day. Five hundred grams of *MCL‐35g* and *CF‐35g* were supplied to mothers/caregivers every two weeks. Infants in study group 2 received MLP as a supplement (*MS‐5g*) and were supplied with 14, five‐gram sachets of *MS‐5g* every two weeks. Mothers were instructed to prepare and feed the study foods a minimum of two times/day in addition to other family foods. For infants receiving *MS‐5g*, mother/caregivers were instructed to sprinkle the one 5 g sachet of *MS‐5g* on the infant's usual foods, a minimum of two times/day. At the bi‐weekly visits, study foods which were left over from the previous visit were weighed and recorded. Adherence to study foods was determined as the disappearance rate of study foods supplied to infants during the study period. Mothers were educated on good hygienic practices and also encouraged to continue breastfeeding. Mothers/caregivers were given plates and spoons for feeding their infants. Study foods were given at no charge to infants.

Morbidity data (diarrhea, vomiting, symptoms of respiratory infections, and fever) were collected every 2 weeks at the same time as a fresh consignment of project foods were delivered to the caregivers.

### 2.7 Endline

At endline, data on anthropometric measurements, 24‐hr recalls and hemoglobin concentration were collected.

### 2.8 Data analyses

Data analyses were done using Statistics package for social sciences (SPSS) version 20, (IBM, USA, 2011). At baseline, characteristics of groups were summarized using descriptive statistics and cross‐tabulations and factor analysis with varimax rotation was used to create a wealth index which was categorized into low, middle, and high. Analysis of covariance (ANCOVA) with post hoc tests for pairwise comparisons was used to determine any significant differences in baseline and endline values for mean change in hemoglobin, weight gain, and length gain among the three study groups at the end of the study. Adjustments were made for wealth index and morbidity (due to group differences) and baseline values whenever possible. Infant sex, maternal education and maternal height and BMI were pre‐specified covariates and controlled for where necessary. All analyses were done first without controlling for covariates and then with covariates. Paired *t*‐test was used to make within‐group comparisons for hemoglobin concentration at baseline and endline. To compare categorical outcomes (anemia, stunting, wasting, and underweight) across the three groups at endline, binary logistic regression was used, whilst relative risks were used for pairwise group comparisons. Chi‐squared tests were used to compare percentages of infants (within group) who were anemic, stunted, wasted, and underweight at baseline to their respective percentages at endline. Occurrence of illness, estimated as percentage of completed visits with reported (by mothers or caregivers of infants) illness during the preceding 14 days, were compared between the groups. Morbidity data were natural log‐transformed before comparison by ANCOVA. The analysis was by intention to treat, that is, children were included in the analyses, whether or not they consumed the full dose of study food during the intervention period.

## 3 RESULTS

### 3.1 Sample characteristics

**Figure 1:**
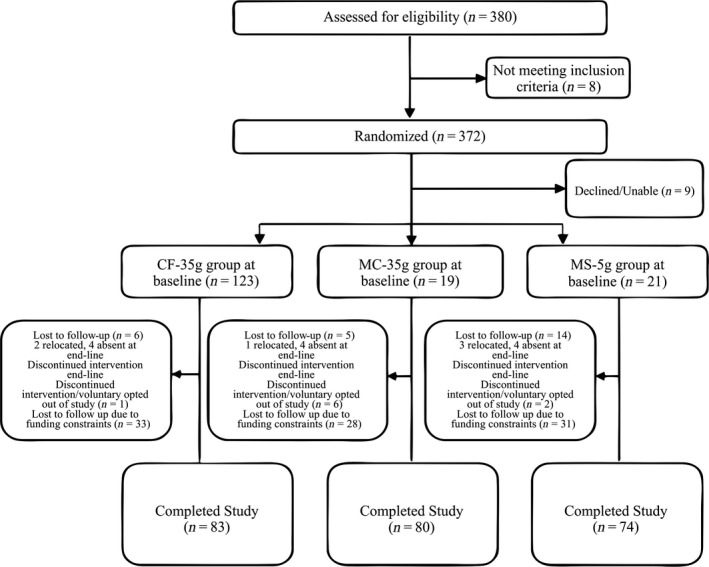
Flow of study participants in the *Moringa* feeding trial

Of 372 eligible infants, 363 were allotted to one of the three study groups. Nine declined to participate (Figure 1), the major reasons being parental refusal and anticipated inability to follow through to the end of the study. At baseline, there were more males in the *MS‐5g* group (35.7%) while *CF‐35g* and *MCL‐35g* groups had equal proportion (32.1%), Table 3. Children in *MS‐5g* group were slightly older with an average age of 9.3 ± 1.4 months followed by *MC‐5g* group (9.1 ± 1.4 months). In terms of birth order, the infants averaged 3rd in birth order for all groups. Majority of mothers in all three study groups had education at least up to primary school level, with mothers in CF group (85.4%) having the highest proportion followed by MC group (84.0%). Body mass index was comparable among mothers in all 3 groups, with the *CF‐35g* group having BMI = 18.1 ± 3.2 kg/m^2^ whilst mothers from *MCL‐35g* and *MS‐5g* groups had BMI of 18.0 ± 3.4 kg/m^2^ and (17.9 ± 3. kg/m^2^), respectively. A higher proportion of households in the *CF‐35g* group (43.9%) were in the highest wealth index compared to 27.7% and 28.1% for the *MCL‐35g* and *MS‐5g* groups respectively (Table 3).

**Table 3:** Background characteristics of study participants at baseline

	*CF‐35g*	*MCL‐35g*	*MS‐5g*
	(*n* = 123)	(*n* = 119)	(*n* = 121)
Infant and family characteristics			
Infant sex, male (%)	32.1	32.1	35.7
Infant age (months)	9.0 ± 1.3	9.1 ± 1.4	9.3 ± 1.4
Birth order	2.7 ± 2.0	2.8 ± 1.7	3.1 ± 1.9
Mother's age (years)	26.8 ± 7.7	25.9 ± 6.8	26.8 ± 7.5
Mother's education (%)			
Primary^+^	85.4	84.0	81.8
Mother's weight (kg)	57.0 ± 10.1	55.3 ± 10.7	56.3 ± 10.8
Mother's height (cm)	157 ± 6.3	157.2 ± 5.5	156.5 ± 6.3
Mother's BMI (kg/m^2^)	18.1 ± 3.2	18.0 ± 3.4	17.9 ± 3.2
Household Characteristics			
Number of people in household	6.3 ± 2.6	6.8 ± 3.4	6.6 ± 2.7
Use public water (%)			
Piped/borehole/well	81.3	48.7	41.3
Wealth Index (%)			
Lowest	36.6	29.4	33.1
Second	19.5	42.9	38.8
Highest	43.9	27.7	28.1
Morbidity (%)			
Vomiting	12.2	18.5	9.9
Diarrhea	22.0	22.7	24.0
Cough	31.7	37.0	29.8
Nasal discharge	43.9	39.5	40.5
Fever	35.0	30.3	34.7

*Note*

*CF‐35g—*control; *MCL‐35g—Moringa* with *Weanimix; MS‐5g—Moringa* as Sprinkles.

Some infants were lost to follow‐up or voluntarily discontinued the study as reported in Figure 1. Majority of infants however were dropped from the study (Figure 1) as a result of study funding constraints. Altogether, 273 infants completed the study (Figure 1) and were included in the data analyses. Percentage attrition averaged 30% for each group.

### 3.2 Hemoglobin concentrations

Differences in hemoglobin levels between baseline and endline for infants who completed the study are presented in Table 4. There were no significant differences between the study groups (*p* = 0.33) after controlling for baseline values, morbidity, and wealth index (Table 4). The control group (CF‐35g) had the highest increase in hemoglobin, followed by the MS‐5g (*Moringa* as Sprinkles group). The MCL‐35g group had the least increase in hemoglobin levels (Table 4).

**Table 4:** Comparison of mean hemoglobin change, weight gain, and length gain across the 3 study groups

Outcome variable	*CF‐35g* a (*n* = 83)	*MCL‐35g* a (*n* = 80)	*MS‐5g* a (*n* = 74)	*p* b	*CF‐35g* and *MCL‐35g* c		*CF‐35g* and *MS‐5g* c		*MS‐5g* and *MCL‐35g* c	
					Mean difference	*p*	Mean difference	*p*	Mean difference	*p*
Hemoglobin change (g/dl)	0.43 ± 1.40	0.15 ± 1.22	0.31 ± 1.36	0.33	0.27 (0.17–0.71)	0.41	0.15 (0.34–0.56)	1.00	0.16 (0.30–0.61)	1.00
Weight gain (kg)	0.91 ± 0.42	0.75 ± 0.51	0.85 ± 0.34	0.05*	1.63 (0.00–0.33)	0.05*	0.07 (0.09–0.23)	1.00	0.09 (0.07–0.26)	0.47
Length gain (cm)	4.71 ± 1.17	4.14 ± 1.29	4.65 ± 1.23	0.02*	0.57 (0.04–1.10)	0.03*	0.06 (0.48–0.61)	1.00	0.51 (0.04–1.05)	0.08

*Notes*

*CF‐35g—*control; *MCL‐35g—Moringa* with *Weanimix; MS‐5g—Moringa* as Sprinkles.

^a^Data are mean ± standard deviation. ^b^ *p* values compare all 3 groups using ANCOVA. ^c^Pairwise comparison of study groups using post hoc tests, reported as mean difference, confidence intervals and their *p*‐values.

Significant at *p* ≤ 0.0.

### 3.3 Weight gain and length gain

Weight gain was highest for infants in the CF‐35g (control) group and lowest in the MCL‐35g group (0.91 vs. 0.75 kg), (Table 4). There was a marginally significant difference in weight gain (*p* = 0.05) between the CF‐35g (control) group and the MCL‐35g group (Weanimix with *Moringa*), after controlling for baseline values and wealth index (Table 4). There was however no statistically significant difference in weight gain between infants in the control arm and infants in the MS‐5g (*Moringa* as sprinkles) arm, (0.91 vs. 0.85 kg) or the two *Moringa* groups (0.75 vs. 0.85 kg). Length gain between baseline and endline was greatest in the CF‐35g (control) group when compared to the two other groups (Table 4). The CF‐35g group had significantly higher (*p* = 0.03) length gain than the MCL‐35g group, but not the MS‐5g group (*p* = 1.00), after controlling for baseline values and wealth index. The observed difference between the two *Moringa* groups was also not statistically significant (*p* = 0.08).

### 3.4 Growth

Mean differences in WHO growth indicators were compared at baseline and endline. Generally, there was a decreasing trend in length‐for‐age *z*‐scores (LAZ) and weight‐for‐length *z*‐scores (WLZ) for all groups, and an observed decrease in weight‐for‐age (WAZ) for the MCL‐35g (Weanimix plus *Moringa*) group (Supporting Information Appendix S1). This decrease was however not statistically significant in any of the three groups for any of the growth indicators even after controlling for baseline values, wealth index, infant sex, and maternal height (Supporting Information Appendix S1).

### 3.5 Comparison of prevalence of anemia, stunting wasting, and underweight

Prevalence of anemia, stunting, underweight, and wasting observed in this study are reported in Supporting Information Appendix S3. Comparison of the outcomes at endline revealed that the three groups did not differ significantly in the percentages of children at endline were anemic, underweight, stunted, and wasted. A comparison of baseline and endline outcomes of study infants within groups however revealed that prevalence of anemia had decreased over the study period for all three groups (Supporting Information Appendix S3). This within‐group difference was however only significant for CF‐35g (control) group (baseline = 63.9% and endline = 47.0%, *p* = 0.04). Although not significant, an increasing trend (within group) in prevalence of wasting, stunting, and underweight was observed for all three groups. This trend was more consistent for the MCL‐35g group than for *CF‐35g* group (which increased only for stunting) and MS‐5g group (which increased for wasting and stunting) and *MS‐5g* group (which increased for wasting and stunting).

### 3.6 Morbidity

The occurrence of diarrhea, vomiting, cough, nasal discharge, and fever in the study infants are shown in Table 5. There were significant differences observed between the study groups in all the morbidity outcomes. The *MCL‐35g* group had the highest occurrence of morbidity for all the outcomes, followed by the *MS‐5g* group. The *CF‐35g* group had the least occurrence of illness. The overall occurrence of diarrhea, vomiting, cough, nasal discharge, and fever was 13.3%, 4.09%, 15.07%, 21.46%, and 18.58%, respectively.

**Table 5:** Occurrence of morbidity* and adherence^§^ to study foods among infants who completed the study

	*CF‐35g* (*n* = 83)		*MCL‐35g* (*n* = 80)		*MS‐5g* (*n* = 74)		*P* ^1^
	$\overset{¯}{X}$	Median (range)	$\overset{¯}{X}$	Median (range)	$\overset{¯}{X}$	Median (range)	
Diarrhea	8.89^a^	0.00 (0–60)	17.06^b^	12.50 (0–83)	14.16^a,b^	12.50 (0–67)	0.02
Vomiting	2.66^a^	0.00 (0–43)	6.26^b^	0.00 (0–60)	3.35^a,b^	12.50 (0–80)	0.03
Cough	12.06^a^	12.50 (0–86)	19.42^b^	14.29 (0–80)	13.75^a,b^	12.50 (0–50)	0.04
Nasal discharge	18.14^a^	14.29 (0–86)	26.16^b^	25.00 (0–100)	20.09^a,b^	14.29 (0–100)	0.05
Fever	13.81^a^	12.50 (0–63)	23.24^b^	16.67 (0–100)	19.90^a,b^	14.29 (0–88)	0.02
Adherence	79.99^a^	78.75 (44–100)	62.11^b^	60.51 (27–100)	60.60^b^	61.16 (4–93)	0.00^2^

*Notes*

*CF‐35g—*control, *MCL‐35g—Moringa* with *Weanimix*,* MS‐5g—Moringa* as Sprinkles; Values are mean percentage of visits with reported illness during the preceding 14 days; Measured as disappearance rate of study foods over the 4‐month follow‐up period. ^a,b^Values in the same row with different superscript alphabets are significantly different, *p *≤* *0.05.

*Values are mean percentage of visits with reported illness during the preceding 14 days; ^§^Measured as disappearance rate of study foods over the 4‐month follow up period; ^1^Groups were compared by using ANCOVA with control for maternal education and wealth index; ^2^Groups were compared using ANOVA.

### 3.7 Adherence

Adherence to study foods measured as the disappearance rate is shown in Table 5. There was a significant difference in adherence with the *CF‐35g* group having significantly higher adherence than the two *Moringa* groups. The difference in adherence between the two *Moringa* groups was however not statistically significant.

## 4 DISCUSSION

Findings of this study were contrary to the study hypotheses which predicted that the infants who were fed complementary foods that incorporated MLP would have significantly higher increase in the primary outcome hemoglobin concentration. The mean change in hemoglobin concentration was numerically higher for infants in the control arm (who did not have any MLP), when compared to infants in the two *Moringa* groups, although the differences were not statistically significant. This finding is notwithstanding of the fact that *Moringa* leaves are rich in beta carotene and can thus be utilized as an important source of vitamin A, a vitamin which is useful for facilitating the release of iron from storage sites and thus helping to improve hemoglobin levels (Zongo, Zoungrana, Savadogo, & Traoré, 2013). Several factors may help explain the findings of this study;

Firstly, the 5‐g daily supply of MLP (from MCL‐35g and MS‐5g) added only 1.13 mg of iron to nutrient intakes of infants in those two study groups. This level which translates into 10% of the Recommended Nutrient Intakes (RNIs) for iron for infants aged between six to 23 months is far lower than the WHO's recommendation of nearly 100% of iron requirements for infants aged 6–23 months coming from complementary foods (WHO, 1998). The 5 g daily dose of MLP incorporated in infants’ complementary foods was chosen due to concerns about acceptability of foods that are fortified with higher levels of MLP (Oyeyinka & Oyeyinka, 2018). With the exception of two studies, one unpublished graduate thesis conducted in Tanzania (Andrew, 2010) and a longitudinal study conducted among malnourished children in Burkina Faso (Zongo et al., 2013), the authors are not aware of any published randomized controlled trials that have studied the supplementation of diets of infants in this age group (8–12 months) with MLP. For the Tanzanian study, children, aged from six to 24 months who were patients in a nutrition rehabilitation unit, were randomly allocated to receive either 250 g of maize porridge to which 25 g of MLP was added or 250 g of plain maize porridge daily for three months (two months at the rehabilitation unit and one month at home). The study reported a significant difference in hemoglobin levels among infants at endline with infants who received MLP having higher hemoglobin concentrations (Andrew, 2010). Infants in Tanzanian study received five times more MLP compared to infants in the two *Moringa* groups of this study received who received 5 g of MLP daily. The lower intake of MLP for infants in this study may have accounted for the results obtained. The Tanzania study did not report adherence to study foods but infants were fed under supervision at the rehabilitation unit for two out of the three months that their study lasted. In this study, infants were fed in their homes with no supervision by research staff. Adherence (which was determined as total percent of study food disappeared over the study duration) was 62% and 61%, respectively, for the two *Moringa* groups (MCL‐35g and MS‐5g) and 80% for the control CF‐35g group. The Burkina Faso study (Zongo et al., 2013), similar to the Tanzania study, compared two groups of malnourished children aged between 6 and 59 months; one group received a 10 g daily dose of MLP for 6 months, whilst the control group did not receive any MLP. The average weight gained daily (8.9 ± 4.30 g/kg/day) which was observed in children who were fed the MLP was significantly higher than in children (5.7 ± 2.72 g/kg/day) who did not receive the MLP but there was no significant increase in hemoglobin levels, observed in either group. Zongo et al. (2013) have however stated that their findings might be better interpreted when complemented by an analyses of other parameters of iron deficiency such as transferrin, ferritin serum, and other biomarkers of inflammation. This is because improvement in nutritional status may not necessarily reflect in changes in single biological parameters such as hemoglobin. With respect to the hemoglobin concentrations, the findings of Zongo et al. (2013) are comparable to the findings of this study. It was not possible to ascertain whether infants alone were fed all the study foods that disappeared as they were not monitored by direct observation. Similar to the results of the acceptability trial (Boateng et al., 2018), mothers in the efficacy trial (including mothers in the two *Moringa* groups) expressed largely positive feedback when asked to comment on their children's liking of foods over the study period and their willingness to feed their infants the study foods in the future. Such responses however may not be definitive as respondents might be reluctant to provide negative assessments (Adu‐Afarwuah, Lartey, Zeilani, & Dewey, 2011). A comparison of the 80% adherence observed in the control group who ate Weanimix alone to the adherence obtained for the two *Moringa* groups however indicated that those study foods may not have been very well accepted by the infants who were assigned to receive them, irrespective of the findings of the acceptability trial indicated that the study foods were acceptable in the population (Boateng et al., 2018).

This study's findings with respect to adherence is consistent with reported findings in the literature which indicate that, despite their improved nutritional quality, increasing levels of MLP could reduce the acceptance of MLP‐fortified foods (Oyeyinka & Oyeyinka, 2018). In this study, percent fortification of MLP was 15% compared to the 11% fortification in the Tanzanian study. Percent fortification is not reported in the Burkina Faso study.

Another factor which may have influenced the hemoglobin results was the food given to infants who were in the control arm of this study, whereas both the Tanzanian study and the Burkinabe study fed plain porridges to infants in their control groups, this study fed a cereal‐legume blend (Weanimix). The superiority of cereal‐legume blends in terms of protein and energy densities, compared with maize, millet or sorghum‐only porridges which are less nutritious, (Amagloh et al., 2012) may have accounted for the study findings, where the infants in the control group had better nutritional outcomes comparatively.

The duration of feeding employed in this study may also have accounted for the observed change in the hemoglobin concentration from baseline to endline. The four‐month study duration chosen for this study was informed by the duration of both the Tanzanian study and the Burkina Faso study which lasted 3 and 6 months, respectively. Both studies were conducted among malnourished infants who were patients of rehabilitation units. Given that infants in this study were recruited from a healthy population, an average duration of four months of feeding was considered feasible. However, this duration might have been inadequate to observe any significant results in hemoglobin and growth indices, given that the daily dose of MLP given to infants in this trial was far lower than what was given in the two studies stated.

Bioavailability of iron in MLP also might have been a challenge. A recent animal study conducted by Gallaher et al. (2017) concluded that bioavailability of iron in *Moringa* is low. Gallaher et al. (2017) further suggested that the low bioavailability of iron from *Moringa* is due to its high phytic acid concentration and that the use of dried *Moringa* leaves, to improve iron status might not be successful and may even result in a worsening of iron status. The authors however concluded that processing of *Moringa* leaves to reduce or remove its phytic acid could improve it iron bioavailability (Gallaher et al., 2017).

Occurrence of morbidity reported by mothers and caregivers over the period of the study is another factor that may have influenced the results of this study. Infants in the CF‐35g group had significantly lower reported morbidity (Table 5) and this may explain why their hemoglobin levels improved the most of the three groups, and significantly less infants were anemic at endline in that group. Similarly, it is not surprising that the *MCL‐35*g group which reported significantly higher morbidity compared to the two other study groups had the least observed increase in hemoglobin levels. Worm infestation and infectious diseases such as malaria and diarrhea are associated with low hemoglobin concentrations (Agho, Dibley, D'Este, & Gibberd, 2008). In the Tanzanian study, children were dewormed and treated against malaria and other infections before the intervention begun and this may have positively influenced their hemoglobin concentrations at endline. In this study, mothers and caregivers who reported infant morbidity were referred to the primary healthcare centers for medical care. Although not explicitly reported, infants in the Burkina Faso study might have also benefited from treatment of infections as they were patients of a nutrition rehabilitation unit. This study recruited healthy infants in the community and did not carry out treatment of infections prior to the intervention, thus, it is possible that the presence of sub‐clinical infections may have influenced morbidity and partly accounted for the lack of significance in the results of the hemoglobin concentrations among the groups at endline. It is also likely that MLP supplementation may be more beneficial for severely malnourished infants as was observed in both the Tanzanian and Burkina Faso studies, rather than for healthy infants.

It is not clear why of the three study groups, the MCL‐35g group recorded the highest reported morbidity (Table 5). All foods were hygienically produced and aseptically packaged at the Food Research Institute (FRI) in Accra and samples were taken from batches of prepared study foods regularly for microbiological testing. Indeed, had there been any contamination originating from the food preparation and packaging, there should have been similar levels of morbidity reported in all three groups. One factor that may have contributed to the reported morbidity in the two *Moringa* groups, may be due to the MLP itself. Although there is little published data on adverse reactions to MLP, being a concentrated and fibrous product, which was fed to infants and young children, it may have elicited symptoms of morbidity (particularly diarrhea) which was noted and reported by the mothers/caregivers who participated in the study. This is irrespective of the fact that it was incorporated as small quantities in infants’ complementary foods and mothers were also taught to thoroughly stir/mix as well as cook/heat before feeding to infants. The study in Tanzania reported the mean age of infants was 14 months whilst the mean age was 9 months in this study. The Burkina Faso study reported the age range of infants as 6–59 months, but did not report the mean age of infants. On the average, the older children in the Tanzania study (and possibly the Burkina Faso study) may have been better able to tolerate the MLP and hence, the lower levels of observed morbidity and significantly better nutritional outcomes observed in the *Moringa* groups of those studies.

Another possible explanation for the differences in the morbidity observed may probably have to do with the socioeconomic indicators of the households in the clusters to which the study foods were randomly allocated. A higher proportion of households of infants (81%) in the control group (CF‐35g) used improved sources of drinking water such as standpipes, boreholes/wells compared to 49% and 41% in the MCL‐35g and MS‐5g groups respectively (Table 3). Poor hygiene and sanitation may have played key roles in the levels of morbidity and hence the hemoglobin levels observed in the two *Moringa* arms of the study, at endline. These findings remained the same however, even after controlling for source of drinking water. Supply of clean water and environmental sanitation have been frequently reported in the scientific literature as factors that are associated with hemoglobin levels among the 6–59 months’ age group of infants (Agho et al., 2008). Further explanation for the morbidity findings of this study may be the fact that, infants in the control group had significantly more food (80%) disappearing, compared to the two *Moringa* groups. Thus, it is likely that the control infants were probably better nourished and therefore better able to fight infections when compared to infants in the two *Moringa* groups.

The pattern of weight gain and length gain observed in this study was similar to that observed for the hemoglobin concentration. (Table 4). Contrary to the study hypothesis, the infants in the control arm gained weight and length which was significantly higher at endline, compared to the two *Moringa* groups. However, a comparison of the differences in the WHO growth indicators, weight‐for‐length *z*‐scores (WLZ), length‐for‐age *z*‐scores (LAZ) and weight‐for‐age *z*‐scores (WAZ) (Supporting Information Appendix S1) from baseline to endline showed no statistically significant difference between the three study groups. There were also no significant differences in percentages of infants who were wasted, stunted, and underweight at both baseline and endline across the groups (Supporting Information Appendix S3). This is contrary to findings of the Tanzanian study where, WAZ, WLZ, and MUAC scores improved significantly for infants in the *Moringa* supplemented group when compared to infants in the control group. The LAZ scores however showed no statistically significant difference (Andrew, 2010). For the Burkina Faso study also, MUAC and WAZ also improved significantly among MLP‐supplemented infants. The reasons for the findings of the anthropometric measurements of this study may be similar to what has been discussed for the hemoglobin levels observed. In retrospect, this study design could have been stronger if a fourth study group, comprising of infants who were not fed with any of the study foods, had been included in the study. However due to practical and budgetary reasons, this was not considered acceptable to the community or feasible.

Our study had some limitations. Funding constraints greatly influenced the numbers of infants we could recruit to start with, as well as even the numbers of infants who could complete the study. This high attrition rate consequently resulted in a low power at the end of the study. A further limitation of this study was that it was not possible to mask the fieldworkers who were responsible for delivering the study foods or the mothers who received the foods, to the design of the study. Also, data on use of multivitamin supplements were not collected and hence could not be controlled for in the data analyses. These limitations notwithstanding, one strength of this study was the fairly equivalent numbers of children who completed the study in each of the 3 groups, thus allowing for statistical comparisons.

## 5 CONCLUSION

Findings of our study indicated that feeding infants with complementary foods fortified with a daily dose of 5 g of MLP was not beneficial in significantly improving their hemoglobin concentrations or growth indicators when compared to infants who did not receive any MLP in their complementary foods, after 4 months of feeding. From these findings, it can be speculated that perhaps, the control food, Weanimix, may be nutritionally adequate for infants in the population studied. Further studies are needed to explore especially, the long‐term acceptability of complementary foods fortified with MLP with particular focus on morbidity. The possibility of recruiting a non‐intervention group whose outcome measures will be measured at endline only should also be considered in subsequent studies, as such a group may better help with comparison of outcome measures at endline.

## CONFLICT OF INTEREST

The authors declare that they have no conflict of interests.

## AUTHOR CONTRIBUTION

LB, AO, MA, and MS‐A wrote and implemented the study. LB and WQ collected the data and performed the data analyses. LB drafted the first draft of the manuscript. LB, WQ, AO, MA, and MS‐A contributed to the critical revision and refinement of the draft. All authors read and approved the manuscript.

## ETHICAL REVIEW

The study protocol was approved by the Ethical Review Committee of the Ghana Health Service (GHS‐ERC: 07/09/14), and the Institutional Review Board of the Noguchi Memorial Institute for Medical Research (NMIMR –IRB CPN ‐106/14‐14), University of Ghana. Permission to use the study foods was obtained from the Food and Drugs Authority in Ghana. Written permission to carry out the study in the Upper Manya Krobo District was obtained from the District Health Administration,

## INFORMED CONSENT

Written informed consent was obtained from the parents of each infant.
