# Supplementary material for: Effect of complementary foods fortified with Moringa oleifera leaf powder on hemoglobin concentration and growth of infants in the Eastern Region of Ghana
Source: Food Sci Nutr. 2018 Nov 20;7(1):302–11. doi: 10.1002/fsn3.890 (PMC6341130; doi:10.1002/fsn3.890)
Supplement: Supplementary file 1 [file FSN3-7-302-s001.docx]

Supplementary Appendix 1- Table 6: Differences in anthropometric indicators from baseline to endline among infants who completed the study by group

|  | *CF – 35g^1^* | *MCL – 35g^1^* | *MS – 5g^1^* |
| --- | --- | --- | --- |
|  | **n=83** | **n=80** | **n=74** |
| Mid Upper Arm Circumference (MUAC) [cm] | | | |
| Baseline | 13.8±1.1 | 14.3±1.2 | 13.7±1.1 |
| Endline | 13.9±0.9 | 14.2±1.1 | 13.9±0.9 |
| Difference | 0.1±0.8 | -0.1±0.8 | 0.2±0.7 |
| Head Circumference (HC) [cm] | | | |
| Baseline | 43.2±1.7 | 43.5±1.7 | 43.2±1.8 |
| Endline | 43.5±1.5 | 43.7±1.5 | 43.7±1.5 |
| Difference | 0.4±0.7 | 0.2±0.9 | 0.5±0.7 |
| Weight for Age z-scores (WAZ) | | | |
| Baseline | -0.9±1.1 | -0.5±1.1 | -0.9±1.1 |
| Endline | -0.9±0.9 | -0.7±1.1 | -0.9±1.0 |
| Difference | 0.0±0.5 | -0.2±1.1 | 0.0±0.3 |
| Length for Age z-scores (LAZ) | | | |
| Baseline | -1.3±1.2 | -0.9±1.0 | -1.2±1.1 |
| Endline | -1.4±1.1 | -1.2±1.0 | -1.3±1.0 |
| Difference | -0.1±0.7 | -0.3±0.6 | -0.1±0.5 |
| Weight for Length z-scores (WLZ) | | | |
| Baseline | -0.2±1.1 | 0.0±1.0 | -0.3±1.0 |
| Endline | -0.3±0.8 | -0.2±1.1 | -0.4±0.9 |
| Difference | -0.1±0.9 | -0.2±0.8 | -0.1±0.6 |

*CF-35g*- control, *MCL-35g* – *Moringa* with *Weanimix*, *MS-5g* – *Moringa* as Sprinkles;

^1^Data are mean ± standard deviation.

Supplementary Appendix 2- Table 7 Comparison of mean differences in haemoglobin, weight, and length between the control group and the 2 combined Moringa groups

|  | *CF-35g^1^*  n=83 | *Moringa groups^1^*  n=154 | P^2^ |
| --- | --- | --- | --- |
|  |  |  |  |
| Haemoglobin (g/dL) | 0.50 ± 1.40 | 0.19 ± 1.29 | 0.08 |
| Weight (kg) | 0.92 ± 0.42 | 0.79 ± 0.43 | 0.03* |
| Length (cm) | 4.68 ± 1.67 | 4.39 ± 1.29 | 0.15 |

^1^Data are mean ± Standard deviation; ^2^P values compare the 2 groups using independent t-test,*Significant at p<0.05

Supplementary Appendix 3 – Table 8: Prevalence of anaemia, stunting, underweight and wasting across the 3 study groups

|  | *CF-35g^1^*  (n=83) | *MCl-35g^1^*  (n=80) | *MS-5g^1^*  (n=74) | *P^2^* | *CF-35g* and *MCl-35g^3^*  RR (95% CI) | *p* | *CF-35g* and *MS-5g^3^*  RR (95% CI) | *p* | *MCL-35g* and *MS-5g^3^*  RR (95% CI) | *p* |
| --- | --- | --- | --- | --- | --- | --- | --- | --- | --- | --- |
| Anaemia (Hb< 11g/dL) | |  |  |  |  |  |  |  |  |  |
| Baseline | 53/83 (63.86)* | 44/80  (55.00) | 39/74 (52.70) |  | 1.16 (0.90,1.50) |  | 1.21 (0.93,1.59) |  | - 1. 0.78,1.40) |  |
| Endline | 39/83 (46.99)* | 36/80  (45.00) | 33/74 (44.59) | 0.95 | 1.04 (0.75,1.46) | 0.46 | 1.05 (0.75,1.48) | 0.44 | 1.01 (0.71,1.43) | 0.54 |
| Stunting (LAZ< -2 SD) | |  |  |  |  |  |  |  |  |  |
| Baseline | 20/83 (24.10) | 9/80  (11.25) | 19/74 (25.68) |  | 2.14 (1.04,4.42) |  | 1.05 (0.75,1.48) |  | - 1. 0.21,0.91) |  |
| Endline | 24/83 (28.92) | 18/80  (22.50) | 21/74 (28.38) | 0.59 | 1.29 (0.76,2.18) | 0.23 | 1.02 (0.46,1.37) | 0.54 | 0.79 (0.46,1.37) | 0.26 |
| Underweight(WAZ< -2 SD) | |  |  |  |  |  |  |  |  |  |
| Baseline | 10/83 (12.05) | 7/10  (8.75) | 13/74 (17.57) |  | 1.38 (0.55,3.44) |  | 0.69 (0.32,1.47) |  | - 1. 0.21,1.18) |  |
| Endline | 8/83  (9.64) | 10/80  (12.5) | 14/74 (18.92) | 0.23 | 0.77 (0.32,1.85) | 0.37 | 0.51 (0.23,1.15) | 0.08 | 0.66 (0.31,1.40) | 0.19 |
| Wasting (WLZ< -2 SD) | |  |  |  |  |  |  |  |  |  |
| Baseline | 3/83  (3.61) | 1/80  (1.25) | 5/74  (6.76) |  | 2.89 (0.31,27.22) |  | 0.54 (0.13,2.16) | 0.30 | 0.19 (0.02,1.55) |  |
| Endline | 1/83  (3.75) | 3/80  (3.75) | 2/74  (2.70) | 0.56 | 0.32 (0.03,3.03) | 0.30 | 0.45 (0.04,4.02) | 0.46 | 1.39 (0.24,8.07) | 0.54 |

*CF-35g*- control, *MCL-35g* – *Moringa* with *Weanimix*, *MS-5g* – *Moringa* as Sprinkles^1^Data are reported as*n/N (%)* of participants.,^2^P values compared all three groups at endline with logistic regression., ^3^Pairwise comparison of study groups using relative risks and 95% confidence intervals [RRs (95% CIs)] with their p-values; *Significant at p < 0.05 Comparing proportions of outcomes within groups, at baseline and endline were done using chi-sqaure tests.
